# Predictors and consequences of rural clients’ satisfaction level in the district public-private mixed health system of Bangladesh

**DOI:** 10.1186/s41256-017-0052-9

**Published:** 2017-11-02

**Authors:** Ashim Roy, Trudy van der Weijden, Nanne de Vries

**Affiliations:** 1 Department of Health Promotion, School CAPHRI, Faculty of Health, Medicine and Life Sciences, Maastricht University, Maastricht, the Netherlands; 2 Department of Family Medicine, School CAPHRI, Faculty of Health, Medicine and Life Sciences, Maastricht University, Maastricht, The Netherlands

**Keywords:** Clients’ satisfaction, Public-private mixed health system, Service quality, Utility value, Clients’ reaction, Bangladesh

## Abstract

**Background:**

We investigated predictors of the rural clients’ satisfaction level (CSL), and interlinks between perceived specific service quality (PSSQ), perceived utility value (PUV), CSL, and clients’ reactions (CR) towards current and future utilization of providers and facilities in the public-private mixed health system of Bangladesh.

**Methods:**

A quantitative study using interviewer-administered questionnaire was conducted among 400 rural patients. CSL was measured both directly and indirectly. Clients’ opinions of PSSQ relating to healthcare structure and process features were measured for indirectly assessing their satisfaction. PUV and CR were also measured indirectly. 5-point Likert scales were used to measure PSSQ, PUV, CSL and CR. Multiple regression and mediation were the models.

**Results:**

Clients’ satisfaction was low in both health sectors with significantly lower in the public than private sector. Accessibility (financial) predicted commonly high variations in CSL both in the public (18.2%) and private sectors (25.0%). Availability predicted incomparably highest variations in CSL in the public sector (34.6%). Structural factors predicted higher variations in clients’ satisfaction in the public sector, which in the private sector were service process-features. Clients’ reaction was the ultimate outcome of PSSQ mediated through PUV and CSL. PUV mediated the effects of PSSQ on clients’ reaction stronger than CSL.

**Conclusion:**

Financial accessibility is a crucial risk of impoverishment in both public and private sectors. Both structural and process features of healthcare are in ample needs for addressing existing low satisfaction in patients in rural Bangladesh.

**Electronic supplementary material:**

The online version of this article (10.1186/s41256-017-0052-9) contains supplementary material, which is available to authorized users.

## Background

‘Clients’ satisfaction’ is of central interest for sustainability in the competitive market economy worldwide [1]. The emerging market economy is directly related to the privatization and marketization of the health care sector. In a market, clients’ satisfaction is a complex phenomenon often influenced by clients’ perception of the quality of goods or services in terms of extrinsic (e.g. brand, marketing) and/or intrinsic (e.g. contents) features [2, 3]. However, clients’ (i.e. patients’) satisfaction in the healthcare market is a relatively more complex and controversial issue than in other markets because of its loose link to technical quality of health services [4]. Although Robinson suggested that patient-centered care is the essence of clients’ satisfaction in healthcare [5], Zeckhauser & Sommers argued that it may raise tension and mutual frustration because of the gaps between needs and demands [6]. Further, Crow et al. highlighted that individual patients’ socio-demography with health conditions and choices are also linked to their satisfaction level [7]. The debate on clients’ satisfaction has become further complicated in the public-private mixed out-of-pocket payments (OPP) model of health systems, in which individuals or households have to pay either fully or a large part of healthcare costs regardless of affordability. The OPP model health system is common in developing countries and a potential barrier to clients’ satisfaction as well as to poverty reduction [8].

While clients’ satisfaction is associated with positive social and economic outcomes [9], their dissatisfaction may lead to ill health, economic loss, and mistrust between client and provider [10]. Thus, clients’ satisfaction has been identified as a common health, economic and political interest in global health systems and has received substantial attention of policymakers, researchers and academicians over decades.

Among the four constructs of the patients’ satisfaction model of Choi et al. [11], perceived service quality was suggested as the prime cognitive construct influencing perceived utility value (another cognitive construct), satisfaction (an affective construct) and ultimately clients’ reactions (a conation) in terms of adherence and ratings to the current treatment and provider, and repurchase intention. Utility value is the clients’ perceptions of benefits compared to sacrifices [3, 7]. A widely used definition of clients’ satisfaction is the extent to which the service quality meets clients’ expectations. Thus, if expectations are greater than the perceived service quality, then clients’ dissatisfaction occurs; this is the key concept of SERVQUAL model [3]. Expectation is often an outcome of prior experience but also of non-experiential information such as word-of-mouth communication (WOM) and publicity [12].

**Fig. 1:**
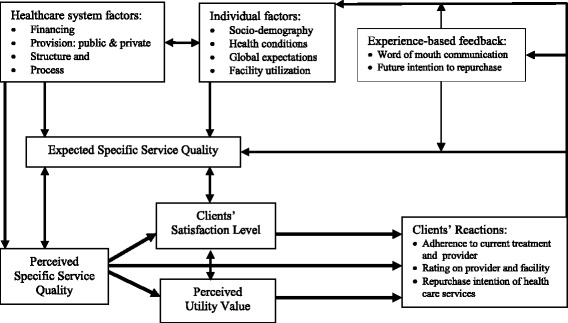
Modified conceptual framework of clients’ satisfaction with health care; Sources: [7, 11, 14]

Concepts and approaches of measuring clients’ satisfaction differ among researchers. A common approach is to measure clients’ satisfaction indirectly through assessing their expectations and perceptions of health service quality. Again, due to the limits of health outcomes to measure service quality, often healthcare structure and process features are assessed as indirect indicators of quality [7, 13]. Also, a combined direct and indirect measurement of satisfaction is evidenced for investigating interrelationships among clients’ perception of service quality, utility value, clients’ satisfaction and their ultimate reactions to health care [11]. Figure 1 illustrates the conceptual model of the present study.

In Bangladesh, there is a clear financing distinction between the public and private health sector. Financing in the public health sector is tax and donor funded, that in the formal private sector is market-based [15]. Only public doctors are entitled as health service providers in the public sector and almost all of them are also involved in the private sector as dual-practitioners. Thereby, dual-practitioners and private-only doctors are the care providers in the private sector. In the public sector, clients pay fixed user fees for defined health services such as diagnostics, but other services e.g. surgeries, bed, foods and available medicines are free. Oppositely, in the private sector, clients usually pay fee-for-services for ambulatory cares directly to the private practitioners and for indoor cares they either pay to the facility owners or to the doctors, mostly specialist dual-practitioners.

Private health expenditure is the larger part, nearly 62% of total health expenditures, of which 96.6% is households’ out-of-pocket payments [16]. Although the costs of the available public health services are far lower than in the private sector, nearly 70% of all clients and even 75% of rural clients seek healthcare from the private sector [15, 17]. Notably, nearly 72% of over 151 million population of Bangladesh are rural dwellers [18], of which 35.2% are below the poverty line [19]. Thus, the burden of healthcare costs on rural people is a major development concern in Bangladesh.

Therefore, identification of the influence of healthcare structure and process-related factors on clients’ satisfaction is essential to limit the impending health and economic threats in this public-private mixed OPP-model health system. This study aimed: to identify the key influences of clients’ expectations and utilization of the district public and private health facilities; to identify the rural clients’ global expectations to the district public and private health sectors; to detect the key factors influencing expected and perceived specific service quality as the predictors of clients’ satisfaction in the public and the private health sector, and to assess the relationships between perceived specific service quality, perceived utility value, satisfaction level, and reactions of the clients in the district health system.

## Methods

### Study design and settings

A quantitative questionnaire study was conducted in Joypurhat district of Bangladesh. Joypurhat was selected purposively because of the following reasons: firstly, the district’s socio-demographic situations match the country’s rural settings. Secondly, the public health system performance of Joypurhat has been recognized by the health ministry as one of the best among all 64 districts for the past eight consecutive years [20]. However, performance of the public health facilities is usually assessed comparing service quantity to a global budget. A system of assessing service quality has yet to be developed; thus, findings of this study would be a benchmark of health service quality and clients’ satisfaction as well for other districts of the country.

Out of a total 913,768 population in the district, nearly 85% are rural residents [21] Joypurhat consists of 5 upazillas (i.e. sub-districts). A 150-bedded district hospital (DH) is the secondary level public health facility. An ‘upazilla health complex’ (UHC) with 50 beds serves as a referral facility of the primary health care (PHC) system of each upazilla. General Physicians and Specialist Doctors are the key service providers of the DH and UHCs. There were fourteen registered private clinics in the district [20].

### Study population

The rural patients/clients of Joypurhat district were the target population. General physicians (GPs), internal medicine and general surgery practices usually serve nearly an equal proportion of male and female patients; therefore, those three departments were selected. Presence of qualified doctors along with outpatient and inpatient services was considered for the inclusion of the public and private facilities. Accordingly, among the public facilities, the district hospital and four UHCs were selected. In the private sector, ten out of fourteen registered clinics had private practice facilities for GPs, internal medicine specialists and general surgeons along with indoor services; thus, these were approached to participate. Patients of providers who had at least five years of professional experience, aged ≥18 years with good physical and mental fitness to provide valid consent were included.

### Sample size and sampling strategy

A sample size of 400 was estimated using the published sampling table of Israel for an infinite population, 95% confidence interval, ±5% precision and 50% degrees of variability [22]. A convenience sampling method was used to select the rural clients from mixed rural-urban crowds. An equal number of clients were sampled from the public and private health facilities (*n* = 200 each). Clients of outpatient and inpatient departments were also equal (*n* = 200 each). An equal number of clients were sampled from the public primary and secondary level facilities (*n* = 100 each).

The ‘probability proportional to size’ sampling method was used to estimate the study units of individual facilities and providers. The total number of registered patients of the sample UHCs in 2015 was used as the sampling frame. No sampling frame was needed for the single district hospital. For the private indoor patients, immediate past one month records of the admitted patients who were treated by the selected doctors were used as the sampling frame and that for the private outdoor patients, seven days records were used. Nearly equal numbers of patients of the selected public doctors were sampled since no doctor-specific patient records were available.

### Data collection tools, techniques and quality control

An interviewer-administered questionnaire was developed containing mostly closed quantitative items. A few qualitative open-ended items also were included; for example, acceptable and experienced approximate waiting time, and sources of health care expenses. Items were mainly based on previous studies (e.g., [7, 11, 14]). The authors initially drafted the tool in English through discussions. The draft questionnaire was then discussed with a panel of seven senior local doctors (public *n* = 4; private *n* = 3) who confirmed the usability of items based on their local contextual experience. Following full consensus on the draft questionnaire, it was translated into local Bengali language. The Bengali version was then again distributed to the doctors and with a few minor changes in wording, it was accepted for piloting. A piloting was conducted with 18 rural clients, which confirmed the instrument’s usability as well as skills of the research assistants. Equal numbers of clients were recruited from public and private sectors (*n* = 9 each) with an equal distribution of outpatient and inpatients of GP, medicine and surgery disciplines including nearly two-thirds (*n* = 11) male clients. All closed items used five-point Likert scales (1 = strongly disagree, 2 = disagree, 3 = neither agree nor disagree, 4 = agree, 5 = strongly agree). No further adaptations were needed. The questionnaire is uploaded in Additional file 1. The following were the key domains, variables and items of the tool:

#### Influences of rural clients’ expectations and utilization of health facilities

A semi-structured multi-response scale was used to explore any effects of the clients’ past experience, WOM-communication, and external communication (e.g. publicity; signboard degrees) on their expectations and utilization of current providers. Clients’ global expectations were investigated using an open-ended scale.

#### Expected and perceived specific service quality

Expected specific service quality (ESSQ) and perceived specific service quality (PSSQ) were measured using the following identically matched eight variables with twenty one items of the two key domains, i.e. structural and process factors:

### Structure related variables and items:

*Tangibility*: outlook of infrastructure, equipment, and records, and cleanliness; *Availability*: supply of drugs and diagnostic tests; *Accessibility* (financial): ability to pay the costs of consultancy, medicines, and diagnostics.

### Process related variables and items:

*Responsiveness*: availability of doctor, and promptness of service delivery; *Reliability*: doctors’ skills, rationale of the advised drugs, and diagnostic tests; *Empathy*: doctors’ attentiveness to patients’ problems, concerns of clients’ financial situation, and mental supports; *Communication*: explanation of diagnosis, treatment plan, and prescription; *Courtesy*: respect to clients, and maintenance of privacy.

#### Perceived utility value

Three items were used: I feel physically well after treatment, I appropriately invested money for treatment, and the quality of services was worth more than what I paid.

#### Direct measure of clients’ satisfaction level

Three items were used: how satisfied were you with the treatment, how satisfied were you with overall dealings of the doctor, and how satisfied were you with the overall services of the hospital/clinic.

#### Clients’ reaction

Four items were queried: I shall follow the current treatment, I shall recommend others to my doctor, I shall recommend others to use this facility, and I shall use this service if I need.

Three teams each consisting of one male and one female qualified medical assistant with working experience in NGO health projects collected data. All research assistants were given one day intensive training on data collection with focuses on to reduce information bias and effective communication. The research assistants invited target clients for interviewing while they were either visiting the selected doctors or admitted in sample facilities. Data was collected at least after one to no later than three weeks from the date of the last visit to the doctors or discharge from sample facilities. Respecting clients’ choice, data were collected mostly at home and in facilities. Informed consent was taken from all respondents. Confidentiality and privacy were maintained. Each interview took an average of 40 min. Two computer experts entered data and cross-checked each other’s work regularly; also the Principal Investigator checked a random sample. Data was collected during August and October 2016.

### Statistical analysis

SPSS (version 21) was used for data processing and analysis. Means and standard deviations of all variables were computed. As recommended by Nunnally [23], Cronbach’s α statistic was used to assess the internal consistency of different scales. Overall internal consistency of the data was acceptable. Cronbach’s α of ESSQ and PSSQ variables are presented in Table 1. Alpha statistics for CSL, PUV, and CR were 0.67, 0.65 and 0.67 respectively.

**Table 1:** Cronbach’s Alpha of the variables of PSSQ and ESSQ domains

Variables	Cronbach’s α		Items
	PSSQ	ESSQ	
Tangibility	0.66	0.81	4
Availability	0.69	0.73	2
Accessibility	0.69	0.67	3
Responsiveness	0.79	0.81	2
Reliability	0.67	0.80	3
Empathy	0.66	0.62	3
Communication	0.77	0.72	2
Courtesy	0.71	0.80	2

*Notes*: *PSSQ* The perceived specific service quality, *ESSQ* Expected specific service quality

Any significance differences in gap scores in means of PSSQ and ESSQ variables, and mean scores of PUV, CSL and CR in the public and private sectors were tested using independent-samples t-test. Chi-squared test was used finding any differences in the public and private clients’ global expectations. Multiple regression models were used identifying predictors of CSL. The interrelationships between PSSQ, PUV, CSL and CR were assessed in simple and multiple mediation models.

Risk of biases relating to outliers and assumptions were tested. Normality of residuals was tested using histograms and P-P plots confirmed. Linearity and homoscedasticity were checked with scatterplots of standardized residuals against standardized predicted values and also partial plots. Leverage value and Cook’s distance statistics were used to test outliers for predictors and influential outliers [24]. Variance inflation factor and tolerance statistic were used to test multicollinearity [25]. Finally, cross-validity predictive power of the model was tested using Stein’s formula [24]. No problems became apparent. The order of block entry in the hierarchical regression approach was based on the gap-scores between the corresponding PSSQ and ESSQ variables; variables with the greater negative ga*p*-values were entered earlier than smaller ones.

The ‘causal steps strategy’ of Baron & Kenny [26] was used to test individual paths in the mediation model. As described by Preacher & Hayes, the Sobel test statistics, i.e. the estimates of the unstandardized indirect effects and their 95% bias corrected and accelerated bootstrap confidence intervals along with *p*-values, were used to assess mediation [27]. The total indirect effect of multiple mediation was calculated by adding the specific indirect effects of PSSQ on CR via PUV and CSL. The kappa-squared (*k* ^*2*^) was used to assess the size of the indirect effects in simple mediation models [28]. Missing data were analyzed using the ‘exclude cases listwise’ option of SPSS. A *p*-value <.05 was considered as significant.

## Results

### Sample characteristics

To interview 400 rural clients, a total of 497 were approached. Extra approaches were required because of following reasons: clients were absent at the given addresses (*n* = 11), interviews were postponed due to clients’ cognitive problems (*n* = 13), refusal of interview (*n* = 24), and clients outside of the study area (*n* = 49). Patients of a total 37 doctors (public *n* = 21; private *n* = 16) of all the sample public and private facilities participated. The ratio of GPs to Specialists in the public sector was 17:4, which in the private sector was 9:7. Of the 16 private sector doctors, the ratio of private-only to dual-practitioners was 6:10. The ratio of patients with acute to chronic disorders in the public and private sector were 84:116 and 111:89 respectively. Patients of wide socio-demographic characteristics were selected (Table 2).

**Table 2:** Socio-demographic characteristics of the full sample, public and private clients

Variable categories	Full sample (*n* = 400)	Public (*n* = 200)	Private (*n* = 200)
	Number (%)		
Age (in years)			
18 - < 30	81 (20.25)	35 (17.5)	46 (23.0)
30 - < 40	84 (21.0)	46 (23.0)	38 (19.0)
40 - < 50	109 (27.25)	60 (30.0)	49 (24.5)
50 - < 60	64 (16.0)	28 (14.0)	36 (18.0)
> 60	62 (15.5)	31 (15.5)	31 (15.5)
Sex			
Male	218 (54.5)	110 (55.0)	108 (54.0)
Female	182 (45.5)	90 (45.0)	92 (46.0)
Marital Status			
Married	309 (77.25)	157 (78.5)	152 (76.0)
Unmarried	32 (8.0)	11 (5.5)	21 (10.5)
Widowed	18 (4.5)	10 (5.0)	8 (4.0)
Others	41 (10.25)	22 (11.0)	19 (9.5)
Educational status			
Illiterate	91 (23.0)	61 (30.5)	30 (15.0)
Primary level	132 (33.0)	81 (40.5)	51 (25.5)
High school level	112 (28.0)	44 (22.0)	68 (34.0)
Above high school level	65 (16.0)	14 (7.0)	51 (25.5)
Monthly income (Taka)			
< 3000	26 (6.5)	23 (11.5)	3 (1.5)
3000 - < 7000	155 (38.75)	103 (51.5)	52 (26.0)
7000 - < 15,000	130 (32.5)	54 (27.0)	76 (38.0)
> 15,000	89 (22.25)	20 (10.0)	69 (34.5)
Occupation			
Housewife	112 (28.0)	52 (26.0)	60 (30.0)
Farming	128 (32.0)	73 (36.5)	55 (27.5)
Day-labour	36 (9.0)	27 (13.5)	9 (4.5)
Business	53 (13.25)	17 (8.5)	36 (18.0)
Official job	29 (7.25)	8 (4.0)	21 (10.5)
Others	42 (10.5)	23 (11.5)	19 (9.5)
Housing condition			
Muddy wall with grass-shade	85 (21.25)	49 (24.5)	36 (18.0)
Muddy wall with tin-shade	89 (22.25)	48 (24.0)	41 (20.5)
Brick-wall with tin-shade	166 (41.5)	77 (38.5)	89 (44.5)
Concrete building	60 (15.0)	26 (13.0)	34 (17.0)

### Influences of clients’ expectations and health facility utilization

Whereas cheap healthcare costs and geographic accessibility were the factors unique for the public clients’ expectations and facility utilization, which in the private clients were external communications, specifically publicity. There were significant differences in effects of ‘WOM-communication’ and ‘past pleasant experiences’ on constructing the public and private clients’ expectations and choice of utilizing facility with higher odds ratios for the private clients (Table 3).

**Table 3:** Frequencies of the factors influence public and private clients’ expectation and utilization of health facilities with chi-square statistics

Factors	Public *n* = 200 (%)	Private *n* = 200 (%)	*Χ* ^2^; *p*-value; odds ratio (*n* = 400; df = 1)
^a^Past pleasant experience	38 (19.0)	60 (30.0)	6.54; .01; 1.83
^a^WOM-communication	52 (26.0)	119 (59.5)	45.85; <.001; 4.18
Cheap health care cost	132 (66.0)	–	–
Near-home health facility	58 (29.0)	–	–
External communication			
Publicity	–	99 (49.5)	–
Signboard degree	–	23 (11.5)	–

*Notes*: (^a^) indicates significant difference; *WOM* Word of mouth, *Χ* ^2^ Pearson’s Chi-Square statistics, *df* Degrees of freedom

### Clients’ global expectations

Availability of common drugs and diagnostics, and free treatment were the most frequent and significantly differentiating global expectations for public clients’ to the health sector. Affordable treatment was a highly frequent global expectation in both sectors but significantly higher in the private clients. Attentiveness and good behavior were markedly high expectations in both sectors without significant differences. However, quick service delivery, providers’ skills and trustworthy treatment were significantly higher expectations in the private than in the public clients (Table 4).

**Table 4:** Frequencies of clients’ global expectations to the health system and providers with chi-square statistics

Domain variable	Public *n* = 200 (%)	Private *n* = 200 (%)	Χ^2^; *p*-value; odds ratio (*n* = 400; df = 1)
Global expectations to health sector			
Free treatment	68 (34.0)	0 (0)	–
^**a**^Affordable treatment	101 (50.5)	131 (65.5)	9.24; .002; 1.86^b^
^**a**^Availability of common diagnostics	109 (54.5)	42 (21.0)	47.76; <.001; 0.22^c^
^**a**^Availability of drugs	97 (48.5)	10 (5.0)	96.57; <.001; 0.05^c^
Cleanliness	23 (11.5)	34 (17.0)	2.48; .11; 1.57^b^
Others	10 (5.0)	14 (7.0)	0.71; .39; 1.43^b^
Global expectations to providers			
Attentiveness	150 (75)	158 (79)	0.9; .34; 1.25^b^
Good behaviour	60 (30.0)	75 (37.5)	2.52; .11; 1.40^b^
^**a**^Skills	19 (9.5)	35 (17.5)	5.48; .02; 2.02^b^
^**a**^Quick service delivery	52 (26.0)	82 (41.0)	10.1; .001; 1.97^b^
^**a**^Trustworthy treatment	24 (12.0)	78 (39.0)	38.37; <.001; 4.69^b^
Others	7 (3.5)	5 (2.5)	0.34; .55; 0.70^c^

*Notes*: *Χ* ^2^ Pearson’s Chi-square statistics, *df* Degrees of freedom

^a^Significant difference

^b^higher odds in the private

^c^higher odds in the public

### Clients’ expectations and perceptions of specific service quality, satisfaction level, utility value and reactions

The overall means of the perceived specific service quality (PSSQ) domain in the full sample, public and private clients were clearly above the neutral value 3 of the five-point scale. The overall means of the expected specific service quality (ESSQ) variables across the client groups were nearly identical but markedly greater than that of the matched PSSQ variables. The overall mean gaps in PSSQ and ESSQ were negative which in the public was nearly 1.5-times larger than in the private clients; the difference was significant [*t* (373.27) = −6.80, *p* = <.001, two-tailed] (Table 5).

**Table 5:** Descriptive statistics including SERVIQUAL gap scores of the public and private samples

Var	Public client (*n* = 200)			Private client (*n* = 200)		
	Mean ESSQ (±SD)	Mean PSSQ (±SD)	Gap score	Mean ESSQ (±SD)	Mean PSSQ (±SD)	Gap score
Tangibility	4.22 (0.33)	3.39 (0.53)	−0.83	4.60 (0.39)	3.82 (0.37)	−0.78
Availability^*^	4.95 (0.18)	3.05 (0.62)	−1.90	4.86 (0.30)	4.21 (0.36)	−0.65
Accessibility^*^	4.92 (0.21)	3.62 (0.41)	−1.30	4.92 (0.19)	3.78 (0.57)	−1.14
Responsiveness^*^	4.55 (0.45)	3.31 (0.99)	−1.24	4.57 (0.44)	3.71 (0.89)	−0.86
Reliability	4.84 (0.31)	3.86 (0.54)	−0.98	4.94 (0.17)	3.98 (0.54)	−0.96
Empathy	4.67 (0.34)	3.68 (0.61)	−0.99	4.71 (0.33)	3.80 (0.45)	−0.91
Communication^*^	4.25 (0.43)	3.22 (0.80)	−1.03	4.26 (0.48)	3.69 (0.72)	−0.57
Courtesy^*^	4.57 (0.47)	3.74 (0.64)	−0.83	4.79 (0.35)	4.26 (0.53)	−0.53
Grand^*^ mean	4.62 (0.18)	3.48 0.52)	−1.14	4.71 (0.18)	3.91 (0.36)	−0.80

Notes: (^*^) indicate variables with significant differences (two-tailed) in PSSQ and ESSQ gap-scores between the public and private sectors based on independent-samples t-tests. *Var* Variable, *PSSQ* Perceived specific service quality, *ESSQ* Expected specific service quality, *SD* Standard deviation, *GS / CSL* Gap score / clients’ satisfaction level; (N.B.: the gap scores are the values produced by subtracting the means of the corresponding variables of the ESSQ domain from that of the PSSQ domain)

Means of clients’ satisfaction level (CSL) in the full sample, public and private clients also were fairly higher than the neutral value, 3.51 (± 0.49), 3.36 (± 0.47) and 3.68 (± 0.44) respectively. By definition the negative gaps between the means of ESSQ and PSSQ variables across the client groups imply dissatisfaction; however, since the overall means of PSSQ and CSL were fairly greater than neutral value, we conjecture it as ‘clients’ low satisfaction level’ (synonymous to CSL). There was significant difference in mean scores of CSL for the public and private sector; *t* (398) = −7.00, *p* = <.001 (two-tailed).

The mean (± SD) of PUV in the full sample was 3.87 (± 0.48), that in the public and private clients were 3.73 (± 0.52) and 4.03 (± 0.37) respectively. In the total sample, perceived benefit (i.e. I feel in better health condition than before) scored notably higher (3.83 ± 0.67) than the neutral value. Client’s reaction (CR) in the full sample, public and private clients scored nearly equally, i.e. 3.68, 3.58 and 3.78 respectively. There were significant differences in mean scores in the public and private sectors for PUV [*t* (355.68) = −6.69, *p* = <.001] and CR [*t* (395.65) = −5.10, *p* = <.001]; two-tailed.

Overall, the sources of covering healthcare costs were as follows: self-financing −66.8%, NGOs (microcredit)/bank loans with interest −6.3%, debts without interest −13.6%, selling household goods −8% and community philanthropy −5.3%. Selling household goods in private clients was nearly twice more frequent than in the public clients; oppositely, community philanthropy was 2.5-times higher in the public than in the private clients.

The acceptable waiting-time (i.e. client-estimated time-gap between enlisting and meeting the doctor for treatment) was nearly identical for public (38 ± 13 min) and private clients (36 ± 12 min). The average waiting-time was also nearly similar in the private (74 ± 53 min) and in the public sector (72 ± 48 min) and both were nearly double the accepted level. Notably, the range of waiting-time was markedly higher in the private (230 min) than in the public sector (175 min). Expected consulting-time in the private was marginally longer than in the public sector, nearly15 (±4) and 12 (±3) minutes respectively. Average perceived consulting-time was nearly 2-times shorter in the public (5.8 ± 2.5 min) than in the private sector (10.6 ± 4.4 min) and both were clearly shorter than the expectation.

### Inferential statistics: Multiple regression of CSL

Normality of residuals, linearity and homoscedasticity were confirmed. The concerns of the influential outliers, independent error and multicollinearity were excluded.

Evaluating the model goodness-of-fit and model parameters.

Overall, *R* ^*2*^ in the public and private clients were notably high (Table 6). *F*-ratios in the final model for the public clients was (*F*_8, 191_ *=* 2.12; *p* = <.001) and for the private clients was (*F*_8, 191_ = 16.75; *p* = <.001) (Additional files 2). The average adjusted values in the public and private clients were very close to the *R* ^*2*^ values (Table 6). This signifies a fairly high cross-validation predictive power of the model.

**Table 6:** Proportions of variations in clients’ satisfaction level by multiple regression models in public and private sectors

PSSQ variable	Public	Private
Availability	34.6%	0.2%
Accessibility	18.2%	25.0%
Responsiveness	7.7%	12.7%
Empathy	5.0%	14.8%
Communication	4.9%	2.3%
Reliability	1.2%	15.8%
Tangibility	0.3%	4.5%
Courtesy	0.3%	2.0%
*Overall cross-validity:*		
Stein’s formula statistic	0.696	0.752
(*R* ^*2*^ value)	(0.722)	(0.773)

Beta-values in the final models of the full sample, public, and private clients were positive indicating the direct relationships among PSSQ variables and CSL. In the private client model, none of the 95% CIs of *b*-values included zero and all *p*-values were <.05 indicating all predictors had statistically significant contributions to the model. However, 95% CIs for availability, responsiveness, tangibility, and courtesy variables in the public clients included zero after entering reliability, tangibility and courtesy in the models 6, 7 and 8 respectively, indicating they do not add to the prediction but overlap with the other variables (Additional files 2).

Whereas availability and accessibility predicted over 73% of the variance explained in CSL for public clients, nearly 91% of explained variance for private clients was explained by accessibility, responsiveness, empathy and reliability. In the public clients, availability was the strongest predictor of CSL, among private clients it was the weakest. Accessibility was an important predictor in both client groups. Remarkably, reliability predicted incomparably high, over 13-times higher variations in CSL in private than in public clients (Table 6).

### Inferential statistics: Mediation

**Fig. 2:**
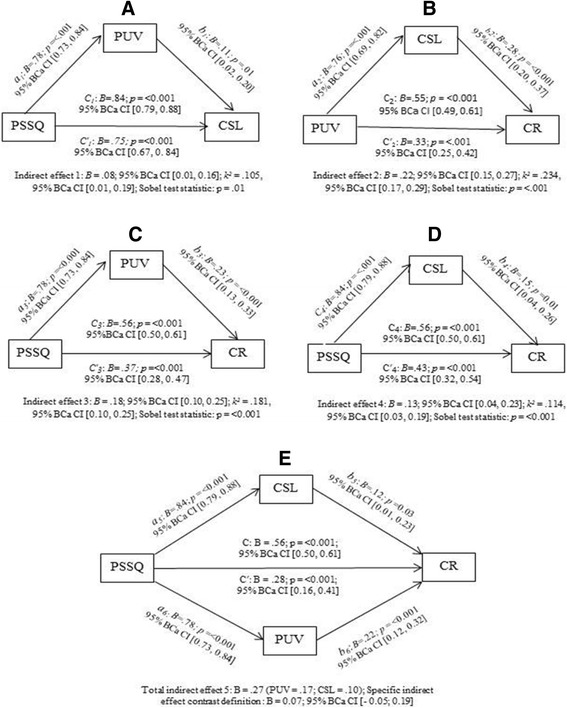
Illustrations of simple mediation (**a**, **b**, **c** & **d** and multiple mediation **e**). Note: 95% BCa CI refers 95% bias corrected and accelerated confidence interval; *k* ^*2*^ stands for Kappa-squared

Significant mediation effects were found in all the simple mediation models (Fig. 2a–d). The indirect effect of the PSSQ on CSL via PUV was small with a *k* ^*2*^ *-* statistic indicating only 10.5% of the maximum possible indirect effect (Fig. 2a) and that for PUV on CR via CSL was markedly large having a *k* ^*2*^-statistic indicating 23.4% of the maximum possible indirect effect (Fig. 2b). The indirect effect of PSSQ on CR via PUV was larger than the indirect effect via CSL with *k* ^*2*^-statistics representing 18.1% and 11.4% of the maximum possible indirect effects respectively. Significant partial mediation was detected in multiple mediation. Of a total indirect effect of PSSQ on CR (27%), PUV contributed nearly twice as much as CSL in the maximum possible indirect effects though marginally significant as the 95% BCa CI of ‘specific indirect effect contrast definition’ included the null value (Fig. 2e).

PSSQ predicted significantly high and nearly equal variations in PUV and CSL, 67.5% and 74.3% respectively which was 49.4% for CR. PUV predicted nearly equal variations of CSL and CR respectively 55.4% and 44.4%.

## Discussion

This study aims to identify key influences of rural clients global expectations and utilization of health facilities, and to determine predictors of their satisfaction level in the public and the private sectors; furthermore, to assess the relationships among the presupposed causal linkage constructs namely: perceived specific service quality (PSSQ), perceived utility value (PUV), clients’ satisfaction level (CSL), and clients’ reactions (CR) in the overall district health system.

### Clients’ global expectations versus expected and perceived specific service quality

Listing the global expectations, respondents did not mention considerable numbers of the items of ESSQ variables we administered and on which they even scored markedly high. This indicates that clients’ expectations are often pre-structured as well as instant context-led construct. While the public clients’ global expectations and selection of facilities mainly were influenced by structural factors (e.g. cheaper and near-home services), which were word-of-mouth communication (a positive reaction), and publicity (a market competition feature) for the private clients. Approximately one-fourth of all clients reported that selection of providers was influenced by past-experiences. These findings indicate that both internal and external factors hold potential to construct clients’ expectations as well as productivity and reputation of health system.

Although clients’ expectations in healthcare are a multifactor construct, its intensity apparently does not differ across clients’ of diverse socio-demographic and individual characteristics and health conditions or structural and process disparities. For instance, the overall means of ESSQ domain are quite high and similar in all groups with markedly small standard deviations (Table 5). This tendency of expectation dynamics was also noticed by Parasuraman et al. [29]; hence, there is a lack of variation regardless of heterogeneity in clients. For this reason, we agree with the critics that the SERVQUAL gap-model is not without risk of statistical unpredictability in assessing diverse clients’ satisfaction in healthcare. However, the ‘perception part’ of the model is useful for indirectly measuring health services quality. We also claim that low quality services; thus, low perceptions rather than high expectations are the key drivers of clients’ low satisfaction, a contradiction to Lewis & Mitchell [30]. Moreover, it is more realistic to improve client’s satisfaction through better services than through lowering expectations.

The overall means of CSL across the clients groups were not perfect; however, still were clearly over the neutral value, which is counterintuitive to the notion of clients’ dissatisfaction with the Bangladesh’s health system. This finding is consistent with other studies (e.g. [10, 31]).

### Predictors of clients’ low satisfaction level (CSL)

In the public sector, structural factors (availability, accessibility and tangibility) predicted nearly three times more of the variations in CSL than process factors (reliability, responsiveness, empathy, communication, and courtesy); in contrast, in the private sector, process features predicted nearly twice as strong as structural factors (Table 6).

*Availability* was the strongest predictor of CSL in the public sector. Low economic status along with high demands of the public clients aggravates the problem of scarce drugs and diagnostics supplies. This finding supports Rahman et al. that sufficient drugs and diagnostics are crucial to improve public clients’ satisfaction [31].

*Accessibility* (financial) was a dominant predictor of CSL in both sectors, although somewhat stronger in the private than in the public sector (Table 6). People having a monthly income <7000 BDT (nearly 88 USD) utilized the public sector over 2-times more often than the private sector (Table 2). However, public clients have to purchase unavailable drugs and diagnostics from the private sector [15]. Moreover, since there is no health insurance system, clients have to bear healthcare costs regardless of their ability to pay. Remarkably, nearly one-thirds of all clients sourced treatment costs by loans with or without interest, selling household goods, and community philanthropy. Although two-thirds of all clients paid healthcare costs by themselves, this does not necessarily confirm affordability. Since only direct healthcare costs were queried, the real situation perhaps was a higher financial burden than was assessed. These findings indicate that financial accessibility in the OPP-model health system with a scarcely resourced public sector and uncontrolled costs in a dominant private market is not only ominously predicting clients’ low satisfaction but also leaving them at risk of poverty. Ample supply with efficient use of resources in the public sector and cost control in the private sector would improve clients’ accessibility and satisfaction, and reduce the risks of a health catastrophe.

*Responsiveness* was a moderately strong predictor of CSL in both sectors though nearly twice as strong as in the public sector (Table 6). Although the perceived average waiting-time was nearly identical in both the sectors, its range in the private was 230 min, i.e. 50 min longer than in the public sector, a counterintuitive impression for the competitive private market. Among the total sample providers only nearly 16% were private-only doctors and the rest were dual-practicing public doctors. Out of the official public working-time, each dual-practitioner runs private practice, often at multiple private facilities and some are intercity practitioners [10], which understandably cause time management problems. It is explicable that the limited specialist dual-practitioners are the monopolists in the private market. As a result, clients’ expectation of quick (ambulatory) services and providers’ interest to exploit income are often mutually exclusive. Noticeably, nearly 87% of surgical treatments (*n* = 76), a major income source in the private sector, were done on the day of admission which was much quicker than in the public sector with an average operation delay of nearly 8.0 (± 3.0) days (*n* = 25). Further, there is no appointment system, especially in the public sector and patients are treated on ‘first-come first-serve’ basis. Therefore, the majority of patients gather at facilities at early hours and eventually many of them meet doctors at late hours causing long mean waiting-times. Furthermore, the current national doctor to population ratio is quite low, nearly 1: 3297 [18], which is even seriously worse in rural areas due to the urban distribution of doctors with a rural to urban ratio of doctors per 10,000 people is 1.1: 18.2 [32, 33]. Thus, low responsiveness is mainly structural in origin.

*Empathy* was also identified as a strong predictor of CSL in the private and nearly 3-times stronger than in the public sector (Table 6). Doctors’ inadequate attentiveness to clients’ problems was also reported by Rahman et al. [31]. Excess workloads were recognized at both the public and private facilities. The role of GPs in the private sector is not established which in turn increases the monopolist specialist doctors’ patient-loads, clearly an expected income interest. These may link to low attention to patients. However, lack of behavioral training of doctors as reported by World Bank [17] may also adversely affect doctor-patient interaction skills; hence, empathy as well. Further, clients’ global expectation of free or cheap treatment remains unmet in both sectors, which is linked to low resource allocation in the public and in the private sector that is not unlikely relating to exploiting profits. Both are counterproductive to the clients’ expectations towards doctors’ consideration for their financial situation.

Reduction of workloads by adapting a hierarchical referral system with GPs as the gatekeeper, which has been proved effective (e.g. in the UK; the Netherlands), would improve responsiveness and empathy as well as clients’ satisfaction. Doctors’ training on ‘behavioural change communication’ would also be effective [17].

*Reliability* was a strong predictor of CSL in the private sector only; however, given the current dual-practicing provision of healthcare, it is unclear why this was not the case in the public sector. The practices of supplier-induced demand (i.e. prescribing irrational drugs and/or diagnostics) for exploiting income, especially in the private market, are of public notions. This may relate to the low reliability rating for the private sector. Health professionals’ skills and moral breakthrough may improve clients’ perception of reliability and satisfaction with the system. Additionally, periodic clinical auditing through an independent multi-actor regulatory body may improve rationale of prescriptions.

*Communication* was a twice stronger but modest predictor of CSL in the public than in the private sector (Table 6). The public doctors are supposed to treat all indoor and outdoor patients that present themselves on any given day. For this, it is not unlikely that they try to treat all the registered client-crowds within the routine official hours (8:00 to 14:30) to save time for private practice. This may explain the reported two times shorter mean consulting time; thus, perception of insufficient communication in the public than in the private clients.

*Tangibility* was a relatively weak predictor of CSL only in the private sector (Table 6). This finding contradicts with the study of [10] that included tertiary level private facilities. It is possible that higher tangibility-related demands of private clients were linked to their better income, housing and education than those in the public clients (Table 2). Additionally, the district level private infrastructures mostly are not constructed as clinics but modified to clinics and visibly inferior to that of public facilities. As a result, the tangible demands of the private clients remain unmet.

### Interrelationships among the constructs of the causal linkage pathway

Perceived specific service quality (PSSQ) being the most influential predictor of perceived utility value (PUV), clients’ satisfaction level (CSL), and clients’ reaction (CR) was reaffirmed as the prime antecedent to the health clients’ satisfaction model that was proposed by Choi et al. [7]. This study also finds that clients’ reaction is the ultimate outcome of PSSQ, PUV and CSL. Statistically significant positive beta-values across all the paths including indirect effects of the simple and multiple mediation models indicate that progress in health services quality would improve clients’ PUV, satisfaction as well as clients’ reaction in favour of adherence to treatment and provider and publicity. Our findings are consistent with other studies (e.g. [11, 34]). However, since the indirect effect and the *k* ^*2*^-statistic of PSSQ on CSL via PUV were the smallest among all the simple mediation models, we suggest a different view of PUV-CSL relationship than other models. For instance, Choi et al. recognized utility value as a cognitive construct [11]; however, it is logical to think that health clients’ satisfaction level may also affect their PUV either as a benefit or a sacrifice. Thus, the PUV is not only a cognitive construct that is strictly antecedent to CSL but in a systemic relation to each other.

Notably, the multiple mediation model (Fig. 2e) showed that PUV mediates the effects of PSSQ on clients’ reaction nearly two times stronger than that of CSL. Moreover, clients’ overall perception of health benefits was scored markedly high, (3.84). This indicates that technical qualities surpass the social aspects of healthcare services. However, the overall findings suggest that both the structural and process features of healthcare are in substantial need of improvement for the rural people’s health and economic safety.

### Strengths and weaknesses

A large number of respondents and nearly equal distributions of participants with diverse socio-demographic characteristics across the public and private sector, female and male, acute and chronic disorders of both out and in-patients departments are the strong points. Face-to-face interview facilitated participation of illiterate respondents and reduced non-response rate. Conducting interviews, mostly at neutral environment, through trained research assistants having community health working experiences reduced the risk of social desirability response bias although central tendency bias, a risk of using Likert scale may not be ignored. The using of a contextually adapted holistic model along with triangulating respondents’ statements of their expectation, perception and socio-economic conditions was also distinctive features of this study.

In order to reduce the risk of changing doctors’ usual manners of dealing with patients as well as to maintain anonymity, we did not inform the doctors whose patients were approached; thus, the doctors were not identified. Alternatively, we identified the patients by discipline (i.e. surgery, medicine and general practice). As a result, we could not control for variations in patients’ satisfaction level across individual doctors. Generalizability of the findings may not be claimed since only one out of 64 districts in Bangladesh was included. However, since the health sector of this district is one of the best performers, our findings would be a benchmark of the clients satisfaction for other districts; thus, would be an asset for policy implications. Doctors’ roles in health clients’ satisfaction is undisputable; however, probing into doctors’ opinion was beyond this study that deserves the need of further research.

## Conclusion

Clients’ low satisfaction in the Bangladesh’s district health system is unquestionable. However, the public clients’ satisfaction is significantly lower than in the private clients. Self-experience, WOM-communication and marketing strategies construct clients’ choices and expectations of utilizing facilities stronger in the private than in the public sector. Prime expectations of the public clients’ are structure-centered, e.g. free treatment and availability of drugs and diagnostics; however, in the private clients those are skills, quick response and trustworthy treatment, i.e. individual provider-concerned. In both sectors, attentiveness was the most frequent process-related global expectation which was affordable treatment among the structural factors with a significantly stronger effect in the private clients.

Improvement of both the structural and process-related service quality factors is pivotal to improve clients’ satisfaction to an optimal expected level. The potential structure and process-related predictors of clients’ satisfaction are clearly under the influence of the ramification of a unique factor- self-economic interest of the key stakeholders such as state’s interests of minimizing healthcare costs, providers’ interest of maximizing income, investors’ interests of increasing profit, and clients’ interests of maximum health benefit with no or minimum payments. In reality, those self-centered interests of the key health actors are mutually exclusive. The growing private health market of the country has been hybridized with the dominant dual-practitioners who are overloaded both in the public and private sectors because of the country’s socio-demography (over-population), and system drawbacks such as under-developed referral system, non-provision of behavioural training and maldistribution of doctors in urban areas. Ultimately, the situation is rendering the country’s majority rural patients at the potential risks of health and economic loss and adversely affecting doctor-patient relationships as well as social capital in healthcare. To improve the situation, policymakers, providers and investors of the health system need to prioritize clients’ health and economic interests.

## Additional files

Additional files 1: The questionnaire of the study. (DOC 223 kb)

Additional files 2: SPSS outputs of multiple regression analysis for predictors of clients' satisfaction level in public and private sectors. (DOCX 37 kb)

## Abbreviations

CR: Clients’ reaction
CSL: Clients’ satisfaction level
DH: District hospital
ESSQ: Expected specific service quality
GPs: General physicians
OPP: Out-of-pocket payments
PHC: Primary health care
PSSQ: Specific service quality
PUV: Perceived utility value
UHC: Upazilla health complex
