# Supplementary material for: Predictors and consequences of rural clients’ satisfaction level in the district public-private mixed health system of Bangladesh
Source: Glob Health Res Policy. 2017 Nov 2;2:31. doi: 10.1186/s41256-017-0052-9 (PMC5683547; doi:10.1186/s41256-017-0052-9)
Supplement: Supplementary file 1 — The questionnaire of the study. (DOC 223 kb) [file 41256_2017_52_MOESM1_ESM.doc]

Appendix 1: Questionnaire for the study entitled – ‘Predictors and consequences of rural clients’ satisfaction level in the district public-private mixed health system of Bangladesh’

Interviewer’s ID No.:……… Serial No.:……….

Date of interview:…………………

Place of interview: Home / hospital / others……………………………….

# Section 1: Respondents background : socio-demographic variables

| Q. No. | Question | Response | Code No. |
| --- | --- | --- | --- |
| 1.1 | How old were you at your last birthday? | Age group in years |  |
|  |  | - 18 - <30 | 1 |
|  |  | - 30 - <40 | 2 |
|  |  | - 40 - <50 | 3 |
|  |  | - 50 - <60 | 4 |
|  |  | - >60 | 5 |
| 1.2 | Gender | - Male | 1 |
|  |  | - Female | 2 |
| 1.3 | What is your marital status? | - Unmarried | 1 |
|  |  | - Married | 2 |
|  |  | - Widowed | 3 |
|  |  | - Others | 4 |
| 1.4 | What is your educational level | - Illiterate | 1 |
|  |  | - Primary level | 2 |
|  |  | - High school level | 3 |
|  |  | - Above high school level | 4 |
| 1.5 | What is the approximate monthly income of you/your family? | - <3,000 BDT | 1 |
|  |  | - 3,000 - <7,000 BDT* | 2 |
|  |  | - 7,000 - <15,000 BDT | 3 |
|  |  | - >15,000 BDT | 5 |
| 1.6 | What is your occupation? | - Farming | 1 |
|  |  | - Business | 2 |
|  |  | - House wife | 3 |
|  |  | - Day-labour | 4 |
|  |  | - Official job | 5 |
|  |  | - Others | 6 |
| 1.7 | Housing condition | - Traditional | 1 |
|  |  | - Brick wall with tin shade | 2 |
|  |  | - Muddy wall with tin shade | 3 |
|  |  | - Concrete building | 4 |

Note: BDT refers Bangladeshi Taka (i.e., local currency).

**Sector 2: Healthcare facility, provision, provider and discipline with health condition identification**

**2.1 Healthcare facility**

| Q. No. | Regarding healthcare provision and facility | Identification | Code No. |
| --- | --- | --- | --- |
| 2.1.1 | Type of provision | - Public | 1 |
|  |  | - Private | 2 |
| 2.1.2 | Level and type of healthcare service | - Public - (primary) upazilla health complex: outpatient | 1 |
|  |  | - Public - (primary) upazilla health complex: inpatient | 2 |
|  |  | - Public - (secondary) district hospital: outpatient | 3 |
|  |  | - Public - (secondary) district hospital: inpatient | 4 |
|  |  | - Private: outpatient | 5 |
|  |  | - Private: inpatient | 6 |

**2.2 Healthcare provider, discipline and disorder**

| Q. No. | Regarding provider and discipline | Remarks | Code No. |
| --- | --- | --- | --- |
| 2.2.1 | Type of provider | - Public | 1 |
|  |  | - Private – dual-practitioner | 2 |
|  |  | - Private-only | 3 |
| 2.2.2 | Practicing discipline | - GP | 1 |
|  |  | - General surgery | 2 |
|  |  | - Internal medicine | 3 |
| 2.2.3 | Type of disorder | - Acute | 1 |
|  |  | - Chronic | 2 |

# Section 3: Factors influence clients’ expectation

**3.1 Why do you choose public health facility**?

| Q. No. | Responses | Code No. |
| --- | --- | --- |
| 3.1.1 | - Past pleasant experience | 1 |
| 3.1.2 | - Recommendation from others | 2 |
| 3.1.3 | - Cost is cheaper than private sector | 3 |
| 3.1.4 | - Health facility is near at home | 4 |
| 3.1.5 | - Others (please specify)………………………………… |  |

| Q. No. | Response | Code. No. |
| --- | --- | --- |
| 3.3.1 | - Past pleasant experience | 1 |
| 3.3.2 | - Recommendation from others | 2 |
| 3.3.3 | - External communication: signboard degrees | 3 |
| 3.3.4 | - External communication: Publicity | 4 |
| 3.3.5 | - Others (please specify)………………………………. |  |

**3.2 Why do you select private healthcare facility / doctor?**

# Section 4: Respondent’s global expectation of healthcare facility and provider (open ended)

| Q. No. | Question | Response | Code No. |
| --- | --- | --- | --- |
| 4.1 | What are your expectations to the healthcare sector? |  |  |
|  |  |
|  |  |
|  |  |
|  |  |
| 4.2 | What are your expectations to the healthcare provider? |  |  |
|  |  |
|  |  |
|  |  |
|  |  |
|  |  |

# Section 5: Expected service quality (specific)

Measuring scale: 1= Strongly disagree, 2= Disagree, 3= Neither agree nor disagree, 4= Agree, 5=Strongly agree

**5.1Tangibility**

| Q. No. | Items | Respondent’s rating | | | | |
| --- | --- | --- | --- | --- | --- | --- |
| 5.1.1 | Hospital / clinic building should be good-looking | 1 | 2 | 3 | 4 | 5 |
| 5.1.2 | Medical equipment should be up-dated | 1 | 2 | 3 | 4 | 5 |
| 5.1.3 | Medical records should be clearly written and attractive | 1 | 2 | 3 | 4 | 5 |
| 5.1.4 | Cleanliness should be maintained | 1 | 2 | 3 | 4 | 5 |

**5.2 Availability**

| Q. No. | Items | Respondent’s rating | | | | |
| --- | --- | --- | --- | --- | --- | --- |
| 5.2.1 | Common drugs should be available | 1 | 2 | 3 | 4 | 5 |
| 5.2.2 | Common diagnostic tests should be available | 1 | 2 | 3 | 4 | 5 |

5.3 Accessibility

| Q. No. | Items | Respondent’s rating | | | | |
| --- | --- | --- | --- | --- | --- | --- |
| 5.3.1 | Cost of drugs should be affordable to general people | 1 | 2 | 3 | 4 | 5 |
| 5.3.2 | Consulting fees should be affordable to general people | 1 | 2 | 3 | 4 | 5 |
| 5.3.3 | Cost of Diagnostics should be affordable to general people | 1 | 2 | 3 | 4 | 5 |

**5.4 Responsiveness**

| Q. No. | Items | Respondent’s rating | | | | |
| --- | --- | --- | --- | --- | --- | --- |
| 5.4.1 | Waiting time should not exceed ……………minutes | | | | | |
| 5.4.2 | Doctor must be available on due time | 1 | 2 | 3 | 4 | 5 |
| 5.4.3 | Quick service should be ensured | 1 | 2 | 3 | 4 | 5 |

**5.5 Reliability**

| **Q. No.** | Items | Respondent’s rating | | | | |
| --- | --- | --- | --- | --- | --- | --- |
| 5.5.1 | Doctor should be professionally competent | 1 | 2 | 3 | 4 | 5 |
| 5.5.2 | Excess diagnostic tests should not be advised | 1 | 2 | 3 | 4 | 5 |
| 5.5.3 | Excess drugs should not be prescribed | 1 | 2 | 3 | 4 | 5 |

5.6 Empathy

| Q. No | Items | Respondent’s rating | | | | |
| --- | --- | --- | --- | --- | --- | --- |
| 5.6.1 | Doctors should be attentive to patient’s problems | 1 | 2 | 3 | 4 | 5 |
| 5.6.2 | Doctors should give mental support to the patient | 1 | 2 | 3 | 4 | 5 |
| 5.6.3 | Doctors should consider patients’ financial ability | 1 | 2 | 3 | 4 | 5 |

**5.7 Communication**

| Q. No | Items | Respondent’s rating | | | | |
| --- | --- | --- | --- | --- | --- | --- |
| 5.7.1 | Doctor should explain the diagnosis and treatment plan | 1 | 2 | 3 | 4 | 5 |
| 5.7.2 | Doctors should clearly explain prescription | 1 | 2 | 3 | 4 | 5 |
| 5.7.3 | Expected minimum consultation time …………… minutes | | | | | |

5.8 Courtesy

| Q.No. | Statement | Respondent’s rating | | | | |
| --- | --- | --- | --- | --- | --- | --- |
| 5.8.1 | Doctors should respect and be friendly to the clients | 1 | 2 | 3 | 4 | 5 |
| 5.8.2 | Doctors should maintain clients’ privacy | 1 | 2 | 3 | 4 | 5 |

**Section 6: Perceived specific service quality**

Measuring scale: 1= Strongly disagree, 2= Disagree, 3= Neither agree nor disagree, 4= Agree, 5= Strongly agree

**6.1Tangibility**

| Q. No. | Items | Respondent’s rating | | | | |
| --- | --- | --- | --- | --- | --- | --- |
| 6.1.1 | Hospital/clinic building is attractive | 1 | 2 | 3 | 4 | 5 |
| 6.1.2 | Medical equipment are up-dated | 1 | 2 | 3 | 4 | 5 |
| 6.1.3 | Medical records are clearly written and attractive | 1 | 2 | 3 | 4 | 5 |
| 6.1.4 | Cleanliness is maintained | 1 | 2 | 3 | 4 | 5 |

**6**.2 Availability

| Q. No. | Items | Respondent’s rating | | | | |
| --- | --- | --- | --- | --- | --- | --- |
| 6.2.1 | Supply of necessary drugs were adequate | 1 | 2 | 3 | 4 | 5 |
| 6.2.2 | Common diagnostic tests were available | 1 | 2 | 3 | 4 | 5 |

**6**.3 Accessibility

| Q. No. | Items | Respondent’s rating | | | | |
| --- | --- | --- | --- | --- | --- | --- |
| 6.3.1 | Costs of necessary medicines were affordable | 1 | 2 | 3 | 4 | 5 |
| 6.3.2 | Consultation fee was affordable | 1 | 2 | 3 | 4 | 5 |
| 6.3.3 | Costs of necessary diagnostic tests were affordable | 1 | 2 | 3 | 4 | 5 |

**6**.4 Responsiveness

| Q. No. | Items | Respondent’s rating | | | | |
| --- | --- | --- | --- | --- | --- | --- |
| 6.4.1 | Waiting time was approximately ………..minutes | | | | | |
| 6.4.2 | Waiting time was acceptable | 1 | 2 | 3 | 4 | 5 |
| 6.4.3 | Doctor was available on time | 1 | 2 | 3 | 4 | 5 |

**6**.5 Reliability

| Q. No. | Items | Respondent’s rating | | | | |
| --- | --- | --- | --- | --- | --- | --- |
| 6.5.1 | My doctor is professionally competent | 1 | 2 | 3 | 4 | 5 |
| 6.5.2 | I think, no excess tests were advised | 1 | 2 | 3 | 4 | 5 |
| 6.5.3 | I think, the prescribed drugs are needed | 1 | 2 | 3 | 4 | 5 |

**6**.6 Empathy

| **Q. No.** | Items | Respondent’s rating | | | | |
| --- | --- | --- | --- | --- | --- | --- |
| 6.6.1 | Doctor was attentive to listen my problems | 1 | 2 | 3 | 4 | 5 |
| 6.6.2 | Doctor provided mental support to me | 1 | 2 | 3 | 4 | 5 |
| 6.6.3 | Doctor was concerned of my financial ability | 1 | 2 | 3 | 4 | 5 |

**6**.7 Communication

| Q. No. | Items | Respondent’s rating | | | | |
| --- | --- | --- | --- | --- | --- | --- |
| 6.7.1 | Doctor clearly explained diagnosis and treatment plan | 1 | 2 | 3 | 4 | 5 |
| 6.7.2 | Doctor clearly explained prescription | 1 | 2 | 3 | 4 | 5 |
| 6.7.3 | Doctor consulted me for a period of…......…..minutes | | | | | |

**6**.8 Courtesy

| Q. No. | Items | Respondent’s rating | | | | |
| --- | --- | --- | --- | --- | --- | --- |
| 6.8.1 | Doctor was respectful and friendly | 1 | 2 | 3 | 4 | 5 |
| 6.8.2 | Doctor maintained my privacy | 1 | 2 | 3 | 4 | 5 |

# Section 7: Client’s perceived utility value

Measuring scale: 1= Strongly disagree, 2= Disagree, 3= Neither agree nor disagree, 4= Agree, 5=Strongly agree

**7.1 Perceived benefits**

| Q. No. | Statement | Respondent’s rating | | | | |
| --- | --- | --- | --- | --- | --- | --- |
| 7.1.1 | I feel better health condition than before | 1 | 2 | 3 | 4 | 5 |
| 7.1.2 | I appropriately invested my money for treatment | 1 | 2 | 3 | 4 | 5 |
| 7.1.3 | The quality of services was worth more than I paid | 1 | 2 | 3 | 4 | 5 |

**7.2 Key sources of healthcare costs**

| Q. No. | Statement | | Respondent’s rating | |
| --- | --- | --- | --- | --- |
| 7.2.1 | Sources of treatment costs | Self-finance | | 1 |
|  |  | I debt money from NGO-microcredit/Bank with interest | | 2 |
|  |  | I debt from others without interest | | 3 |
|  |  | I sold household goods to conduct my treatment | | 4 |
|  |  | Self-financing with community philanthropy | | 5 |

**Section 8: Clients’ reaction to treatment, doctor and facility**

Measuring scale: 1= Strongly disagree, 2 = Disagree, 3 = Neither agree nor disagree, 4 = Agree, 5 = Strongly agree

| Q. No | Statement | Respondent’s rating | | | | |
| --- | --- | --- | --- | --- | --- | --- |
| 8.1 | I shall follow the current treatment | 1 | 2 | 3 | 4 | 5 |
| 8.2 | I would consider this service in future if I need | 1 | 2 | 3 | 4 | 5 |
| 8.3 | I shall recommend others about my doctor | 1 | 2 | 3 | 4 | 5 |
| 8.4 | I shall recommend others to use this facility | 1 | 2 | 3 | 4 | 5 |

**9. Client’s opinion of overall satisfaction level: direct measurement**

Measuring scale: 1= Very unhappy; 2= Unhappy; 3= Neutral; 4= Happy; 5= Very happy

| Q. No | Statement | Respondent’s rating | | | | |
| --- | --- | --- | --- | --- | --- | --- |
| 9.1 | How satisfied were you with the treatment? | 1 | 2 | 3 | 4 | 5 |
| 9.2 | How satisfied were you with overall dealings of the doctor | 1 | 2 | 3 | 4 | 5 |
| 9.3 | How satisfied were you with overall healthcare services of the hospital / clinic? | 1 | 2 | 3 | 4 | 5 |

**10. What was waiting-time in days of performing your operation following admission in hospital / clinic?**

- Same day
- ………..days
