# Supplementary material for: Predictors and consequences of rural clients’ satisfaction level in the district public-private mixed health system of Bangladesh
Source: Glob Health Res Policy. 2017 Nov 2;2:31. doi: 10.1186/s41256-017-0052-9 (PMC5683547; doi:10.1186/s41256-017-0052-9)
Supplement: Supplementary file 2 — SPSS outputs of multiple regression analysis for predictors of clients' satisfaction level in public and private sectors. (DOCX 37 kb) [file 41256_2017_52_MOESM2_ESM.docx]

Appendices 2

(Notes: ΔR denotes changes in *R*², and all adjusted *R*² are the SPSS computed products using Wherry’s formula).

Appendix 1: SPSS outputs of the hierarchical approach of multiple regression analysis - perceived specific service quality variables as the predictors of clients satisfaction level in the public sector.

| Models | Predictors | Unstandardized Coefficients | | Standardized  *β* | *p*-value |
| --- | --- | --- | --- | --- | --- |
|  |  | *β* (95% CI) | Stand. Error |  |  |
| 1 | Constant | 2.00 (1.73, 2.26) | 0.13 | - | <.001 |
|  | Availability | 0.44 (0.36, 0.53) | 0.04 | .59 | <.001 |
| Model 1 Summary: *R* = .588; *R*² = .346; Adjusted *R*² = .343; Δ*R* = 0.346; *F* change (1,198) = 104.84; *P* = <.001 | | | | | |
| 2 | Constant | .53(0.13, 0.94) | 0.04 | - | .009 |
|  | Availability | .28 (0.20, 0.36) | 0.06 | .37 | <.001 |
|  | Accessibility | .54 (0.42, 0.66) | 0.20 | .48 | <.001 |
| Model 2 Summary: *R* = .727; *R*² = .529; Adjusted *R*² = .524; Δ*R* = .182; *F* change (2, 197) = 76.23; *P* = <.001 | | | | | |
| 3 | Constant | 1.07(0.66, 1.47) | 0.04 | - | <.001 |
|  | Availability | 0.13 (0.04, 0.22) | 0.07 | .18 | .004 |
|  | Accessibility | 0.33 (0.20, 0.46) | 0.03 | .30 | <.001 |
|  | Responsiveness | 0.20 (0.14, 0.26) | 0.19 | .42 | <.001 |
| Model 3 Summary: *R* = .778; *R*² = .606; Adjusted *R*² = .599; Δ*R* = .077; *F* change (3, 196) = 38.21; *P* = <.001 | | | | | |
| 4 | Constant | 1.05 (0.67, 1.43) | 0.19 | - | <.001 |
|  | Availability | 0.11 (0.02, 0.19) | 0.04 | .14 | .012 |
|  | Accessibility | 0.27(0.14, 0.39) | 0.06 | .24 | <.001 |
|  | Responsiveness | 0.12 (0.05, 0.19) | 0.03 | .26 | <.001 |
|  | Communication | 0.19 (0.12, 0.26) | 0.04 | .32 | <.001 |
| Model 4 Summary: *R* = .809; *R*² = .654; Adjusted *R*² = .647; Δ*R* = .049; *F* change (4, 195) = 27.34; *P* = <.001 | | | | | |
| 5 | Constant | 0.81 (0.45, 0.18) | 0.18 | - | <.001 |
|  | Availability | 0.07 (0.01, 0.15) | 0.04 | .10 | .053 |
|  | Accessibility | 0.18 (0.06, 0.30) | 0.06 | .16 | .003 |
|  | Responsiveness | 0.08 (0.01, 0.14) | 0.03 | .16 | .018 |
|  | Communication | 0.14 (0.07, 0.20) | 0.03 | .24 | <.001 |
|  | Empathy | 0.25 (0.17, 0.34) | 0.04 | .33 | <.001 |
| Model 5 Summary: *R* = .839; *R*² = 0.704; Adjusted *R*² = .696; Δ*R* = .050; *F* change (5, 194) = 32.63; *P* = <.001 | | | | | |

Continuation of appendix 2

| Models | Predictors | Unstandardized Coefficients | | Standardized  *β* | *p*-value |
| --- | --- | --- | --- | --- | --- |
|  |  | *β* (95% CI) | Stand. Error |  |  |
| 6 | Constant | 0.56 (0.17, 0.96) | 0.20 | - | .005 |
|  | Availability | 0.06 (- 0.01, 0.14) | 0.04 | .08 | .102 |
|  | Accessibility | 0.17 (0.06, 0.29) | 0.06 | .15 | .004 |
|  | Responsiveness | 0.05 (-0.01, 0.12) | 0.03 | .12 | .098 |
|  | Communication | 0.12 (0.05, 0.19) | 0.03 | .21 | <.001 |
|  | Empathy | 0.24 (0.15, 0.32) | 0.04 | .31 | <.001 |
|  | Reliability | 0.13 (0.04, 0.22) | 0.05 | .15 | .005 |
| Model 6 Summary: *R* = .846; *R*² = .716; Adjusted *R*² = .707; Δ*R* = .012; *F* change (6, 193) = 8.09; *P* = <.001 | | | | | |
| 7 | Constant | 0.48 (0.08, 0.89) | 0.21 | - | .020 |
|  | Availability | 0.05 (-0.02, 0.13) | 0.04 | .07 | .179 |
|  | Accessibility | 0.17 (0.05, 0.28) | 0.06 | .15 | .006 |
|  | Responsiveness | 0.04 (-0.03, 0.11) | 0.03 | .08 | .247 |
|  | Communication | 0.11 (0.04, 0.18) | 0.03 | .19 | .002 |
|  | Empathy | 0.23 (0.14, 0.31) | 0.04 | .29 | <.001 |
|  | Reliability | 0.13 (0.03, 0.22) | 0.05 | .15 | .007 |
|  | Tangibility | 0.09 (-0.03, 0.20) | 0.06 | .10 | .148 |
| Model 7 Summary: *R* = .848; *R*² = .719; Adjusted *R*² = .709; Δ*R* = .003; *F* change (7, 192) = 2.10; *P* = <.001 | | | | | |
| 8 | Constant | 0.42 (0.00, 0.84) | 0.21 | - | .046 |
|  | Availability | 0.05 (-0.02, 0.13) | 0.04 | .07 | .180 |
|  | Accessibility | 0.15 (0.04, 0.27) | 0.06 | .14 | .010 |
|  | Responsiveness | 0.03 (-0.04, 0.10) | 0.03 | .06 | .386 |
|  | Communication | 0.11 (0.04, 0.17) | 0.03 | .18 | .003 |
|  | Empathy | 0.22 (0.13, 0.30) | 0.04 | .28 | <.001 |
|  | Reliability | 0.12 (0.03, 0.21) | 0.05 | .14 | .011 |
|  | Tangibility | 0.09 (-0.02, 0.20) | 0.06 | .10 | .134 |
|  | Courtesy | 0.05 (-0.01, 0.12) | 0.04 | .07 | .147 |
| Model 8 Summary: *R* = .850 *R*² = 0.722; Adjusted *R*² = .710; Δ*R* = .003; *F* change (8, 191) = 2.12; *P* = <.001 | | | | | |

Appendix 3: SPSS outputs of the hierarchical approach of multiple regression analysis - perceived specific service quality variables as the predictors of clients satisfaction level in the private sector.

| Models | Predictors | Unstandardized Coefficients | | Standardized  *β* | *p*-value |
| --- | --- | --- | --- | --- | --- |
|  |  | *β* (95% CI) | Stand. Error |  |  |
| 1 | Constant | 2.21(1.85, 2.57) | .18 | - | <.001 |
|  | Accessibility | 0.39 (0.29, 0.48) | .05 | .50 | <.001 |
| Model 1 Summary: *R* = .500; *R*² = .250; Adjusted *R*² = .247; Δ*R* = .250; *F* change (1, 198) = 66.14; *P* = <.001 | | | | | |
| 2 | Constant | 1.12 (0.68, 1.55) | 0.22 | - | <.001 |
|  | Accessibility | 0.33 (0.24, 0.41) | 0.04 | .42 | <.001 |
|  | Reliability | 0.33 (0.24, 0.42) | 0.05 | .40 | <.001 |
| Model 2 Summary: *R* = .639; *R*² = .408; Adjusted *R*² = .402; Δ*R* = .158; *F* change (2, 197) = 52.48; *P* = <.001 | | | | | |
| 3 | Constant | 0.28 (- 0.15, 0.71) | 0.22 | - | .201 |
|  | Accessibility | 0.21(0.13, 0.29) | 0.04 | .28 | <.001 |
|  | Reliability | 0.25 (0.17, 0.33) | 0.04 | .30 | <.001 |
|  | Empathy | 0.42 (0.32, 0.52) | 0.05 | .43 | <.001 |
| Model 3 Summary: *R* = .746; *R*² = .556; Adjusted *R*² = .550; Δ*R* = .148; *F* change (3, 196) = 65.51; *P* = <.001 | | | | | |
| 4 | Constant | 0.55 (0.18, 0.92) | 0.19 | - | .004 |
|  | Accessibility | 0.16 (0.09, 0.22) | 0.03 | .20 | <.001 |
|  | Reliability | 0.15 (0.08, 0.22) | 0.04 | .19 | <.001 |
|  | Empathy | 0.30 (0.21, 0.39) | 0.05 | .31 | <.001 |
|  | Responsiveness | 0.21 (0.16, 0.26) | 0.02 | .42 | <.001 |
| Model 4 Summary: *R* = .827; *R*² = .683; Adjusted *R*² = .677; Δ*R* = .127; *F* change (4, 195) = 78.05; *P* = <.001 | | | | | |
| 5 | Constant | - 0.10 (- 0.51, 0.31) | 0.21 | - | .638 |
|  | Accessibility | 0.14 (0.08, 0.20) | 0.03 | .18 | <.001 |
|  | Reliability | 0.14 (0.08, 0.21) | 0.03 | .17 | <.001 |
|  | Empathy | 0.23 (0.14, 0.31) | 0.04 | .23 | <.001 |
|  | Responsiveness | 0.20 (0.15, 0.24) | 0.02 | .39 | <.001 |
|  | Tangibility | 0.28 (0.18, 0.38) | 0.05 | .24 | <.001 |
| Model 5 Summary: *R* = .854; *R*² = .728; Adjusted *R*² = .721; Δ*R* = .045; *F* change (5, 194) = 32.35; *P* = <.001 | | | | | |

Continuation of appendix 3

| Models | Predictors | Unstandardized Coefficients | | Standardized  *β* | *p*-value |
| --- | --- | --- | --- | --- | --- |
|  |  | *β* (95% CI) | Stand. Error |  |  |
| 6 | Constant | -0.26 (- 0.74, 0.22) | 0.24 | - | .288 |
|  | Accessibility | 0.14 (0.08, 0.20) | 0.03 | .18 | <.001 |
|  | Reliability | 0.14 (0.07, 0.21) | 0.03 | .17 | <.001 |
|  | Empathy | 0.22 (0.13, 0.31) | 0.04 | .23 | <.001 |
|  | Responsiveness | 0.20 (0.15, 0.24) | 0.02 | .39 | <.001 |
|  | Tangibility | 0.27 (0.17, 0.37) | 0.05 | .23 | <.001 |
|  | Availability | 0.06 (- 0.03, 0.16) | 0.05 | .05 | .208 |
| Model 6 Summary: *R* = .855; *R*² = .731; Adjusted *R*² = .722; Δ*R* = .002; *F* change (6, 193) = 1.60; *P* = .208 | | | | | |
| 7 | Constant | -0.30 (- 0.76, 0.16) | 0.23 | - | .197 |
|  | Accessibility | 0.13 (0.07, 0.19) | 0.03 | .17 | <.001 |
|  | Reliability | 0.12 (0.05, 0.18) | 0.03 | .14 | <.001 |
|  | Empathy | 0.20 (0.11, 0.28) | 0.04 | .20 | <.001 |
|  | Responsiveness | 0.15 (0.10, 0.20) | 0.02 | .30 | <.001 |
|  | Tangibility | 0.23 (0.13, 0.33) | 0.05 | .19 | <.001 |
|  | Availability | 0.09 (- 0.01, 0.18) | 0.05 | .07 | .062 |
|  | Communication | 0.13 (0.07, 0.18) | 0.03 | .20 | <.001 |
| Model 7 Summary: *R* = .868; *R*² = .754; Adjusted *R*² = .745; Δ*R* = .023; *F* change (7, 192) = 18.32; *P* = <.001 | | | | | |
| 8 | Constant | -0.47 (- 0.92, - 0.02) | 0.23 | - | .040 |
|  | Accessibility | 0.10 (0.05, 0.16) | 0.03 | .14 | .001 |
|  | Reliability | 0.10 (0.03, 0.16) | 0.03 | .12 | .003 |
|  | Empathy | 0.17 (0.09, 0.26) | 0.04 | .18 | <.001 |
|  | Responsiveness | 0.13 (0.08, 0.17) | 0.02 | .26 | <.001 |
|  | Tangibility | 0.20 (0.10, 0.29) | 0.05 | .17 | <.001 |
|  | Availability | 0.09 ( 0.00, 0.17) | 0.04 | .07 | .053 |
|  | Communication | 0.13 (0.07, 0.18) | 0.03 | .21 | <.001 |
|  | Courtesy | 0.14 (0.07, 0.21) | 0.03 | .17 | <.001 |
| Model 8 Summary: *R* = .880; *R*² = .724; Adjusted *R*² = .765; Δ*R* = .020; *F* change (8, 191) = 16.75; *P* = <.001 | | | | | |
